# Early Surgical Reconstruction Versus Rehabilitation for Patients With Anterior Cruciate Ligament Rupture: A Systematic Review and Meta-Analysis

**DOI:** 10.7759/cureus.43370

**Published:** 2023-08-12

**Authors:** Omar S Dahduli, Abdullah M AlHossan, Mohammed A Al Rushud, Muath M Alneghaimshi, Saad F Alotaibi, Mohammed K AlNour, Abdulrhman H Al Otaibi, Ali AlAseeri, Saud AlBatati

**Affiliations:** 1 Department of Orthopedic Surgery, King Fahad Military Medical Complex, Dhahran, SAU; 2 College of Medicine, Alfaisal University College of Medicine, Riyadh, SAU; 3 Department of Orthopedic Surgery, King Faisal Specialist Hospital and Research Centre, Riyadh, SAU; 4 College of Medicine, Shaqra University, Shaqra, SAU; 5 Department of Orthopedic Surgery, Dammam Medical Complex, Dammam, SAU; 6 College of Medicine, Jouf University, Sakaka, SAU; 7 Department of General Surgery, King Faisal Specialist Hospital and Research Centre, Riyadh, SAU

**Keywords:** orthopedic sports medicine, meta-analysis, anterior cruciate ligament, rehabilitation, reconstruction

## Abstract

Anterior cruciate ligament (ACL) rupture is a common and debilitating knee injury that can significantly impair knee function and stability. The optimal management of ACL injuries remains a topic of ongoing debate, with two primary treatment approaches being surgical reconstruction and adequate rehabilitation. The aim of this study is to compare the knee function and stability outcomes between these two treatment modalities, shedding light on their respective effectiveness.

We utilized Scopus, PubMed, Cochrane Database, MEDLINE, and Web of Science from inception until April 20, 2022. We utilized the Cochrane risk of bias tool for quality assessment. The following outcomes were assessed: Knee Injury and Osteoarthritis Outcome Score (KOOS), International Knee Documentation Committee (IKDC) Subjective Knee score, Lysholm score, the occurrence of the knee giving way, Tegner score, KT1000, Lachman test, pivot shift test, SF-36 score to assess the quality of life, and incidence of reinjury and reoperation.

We included a total of six trials with a population sample size of 691 patients, which were divided into surgical versus non-surgical groups, accounting for 348 and 343 patients, respectively. The pooled estimate demonstrated that the surgical reconstruction was associated with a significant increase in the IKDC score (MD = 7.49 [2.04, 12.94], (P = 0.007)), and KOOS score was significant in the reconstruction cohort (MD = 5.87 [1.64, 10.09], (P = 0.007)). The incidences of reoperation (RR = 0.43 [0.20, 0.91], (P = 0.03)), reinjury (RR = 0.49 [0.27, 0.88], (P = 0.02)), and occurrence of the knee giving way (RR = 0.19 [0.08, 0.49], (P = 0.005)) were significantly decreased in the surgical cohort. There is no significant difference between both cohorts regarding the Lysholm score (1.27 [−1.39, 3.93], (P = 0.35)).

The findings of this comprehensive analysis indicate that early reconstruction does not demonstrate clear superiority over rehabilitation alone in terms of knee function, Lysholm score, and Tegner score among patients with ACL rupture. However, early reconstruction does exhibit a substantial reduction in the incidence of reinjury, reoperation, and knee giving way, suggesting potential benefits in terms of stability outcomes. These results underscore the importance of considering individual patient characteristics and preferences in treatment decision-making.

## Introduction and background

Introduction

One of the primary factors contributing to knee stabilization and function is the anterior cruciate ligament (ACL), which limits anterior tibia movement relative to the femur and aids in tibial rotational stability [1,2]. ACL injuries, often resulting from acute trauma, can be debilitating, particularly in young athletes [3-5]. Knee instability, painful swelling, and associated meniscal injury are key symptoms of ACL injury [6]. Furthermore, ACL injury heightens the risk of knee osteoarthritis (OA), with reported prevalence ranging from 40% to 70% [7,8]. Long-term monitoring of soccer players underscores the importance of managing anterior tibial translation through intensive rehabilitation or ACL reconstruction to curtail meniscal injuries and degeneration [9]. Treatment strategies for ACL injury depend on activity levels, injury types, and instability degree [10]. Patients with low-intensity activity and mild symptoms may respond to physical therapy, while those with severe injuries and pronounced symptoms may require surgery. The KANON trial demonstrated successful non-surgical treatment in around 50% of patients [11,12]. Despite ACL reconstruction offering improved stability and performance, the risk of OA development remains higher compared to an uninjured knee [13]. Enhancing neuromuscular function correlates with better outcomes and OA prevention [14], as muscle power is indicative of OA development; robust physiotherapy and rehabilitation can strengthen knee muscles, reducing OA risk [15]. This meta-analysis aims to evaluate two common treatments, surgical reconstruction and rehabilitation, concerning knee function, OA occurrence, and quality of life.

Methods

We searched PubMed, Scopus, Cochrane Database, Web of Science, and MEDLINE from inception until April 20, 2022, using expert-assisted search strategies with the terms “Surgery,” “Reconstruction,” “Non-surgical,” and “Rehabilitation.”

Database search

We explored PubMed, Scopus, Cochrane Database, Web of Science, and MEDLINE from inception until April 20, 2022. An expert database librarian assisted in performing the ideal search strategy. We used the following terms: “Surgery,” “Reconstruction,” “Non-surgical,” and “Rehabilitation.”

Eligibility criteria

We included all studies that met the following evidence-based PICOS (population, intervention, comparison outcomes, and study design) criteria: (i) population: patients who had ACL injuries; (ii) intervention: ACL reconstruction surgery; (iii) comparison: adequate rehabilitation and physiotherapy; (iv) outcomes: reporting of at least one of the predetermined primary or secondary endpoints; and (v) study design: clinical trials published in peer-reviewed journals. We excluded all studies that met the following criteria: (i) patients who had knee injuries other than specifically ACL injuries, (ii) patients who received mixed surgical and non-surgical interventions at the same time, (iii) studies that had no reliable extraction of data or did not report at least one of our predefined outcomes, and (iv) studies with methodological designs other than clinical trials, such as conference abstracts, animal studies, single-arm investigations, and pharmacokinetic interrogations.

Screening of results

The studies were imported into Excel using the EndNote X8.0.1 version as the first stage in the screening process. The studies in the Excel sheet were then screened for titles and abstracts. The authors then read over the complete texts of the research from the previous step.

Extraction of data

Two independent authors retrieved the data from the involved studies. They took the demographic information about the patients; information about the primary and secondary outcomes, such as Knee Injury and Osteoarthritis Outcome Score (KOOS), International Knee Documentation Committee (IKDC) Subjective Knee score, Lysholm score, probability of the knee giving way, Tegner score, KT1000, Lachman test, pivot shift test, SF-36 score to evaluate the quality of life, and probability of reinjury and reoperation; and finally, the data required for the quality assessment.

Risk of bias assessment

We performed the quality assessment of the involved trials using the Cochrane risk of bias tool. This assessment included adequate blinding, allocation concealment, and randomization of patients through seven domains. The evaluation of domains yielded a high, unclear, or low risk of bias.

Statistical analysis

Using the Mantel-Haenszel method, we examined the dichotomous data and summarized them as risk ratio (RR) with a 95% confidence interval (CI). On the other hand, using the inverse-variance method, we examined the continuous data and summarized them as mean difference (MD) with 95% CI. RevMan Software was used to analyze all the data [16]. To measure the extent of heterogeneity of our pooled outcomes, we employed the chi-square test and I^2^ index. Heterogeneity was determined based on P > 0.1 or I^2^ > 50% [17]. The fixed-effects model was utilized to analyze homogeneous outcomes. Conversely, the random-effects model was utilized to analyze heterogeneous outcomes. The random-effects model is fundamentally based on the inverse-variance methodology, rendering an adjustment to the study weights according to the extent of heterogeneity, among the different intervention effects. We applied Cochrane's leave-one-out sensitivity analysis method in an attempt to resolve the source of heterogeneity, by omitting one study at a time (i.e., zero weight on forest plots) and then recalculating the pooled effect size of the remaining studies.

## Review

Results

Summary of Included Studies

**Figure 1:**
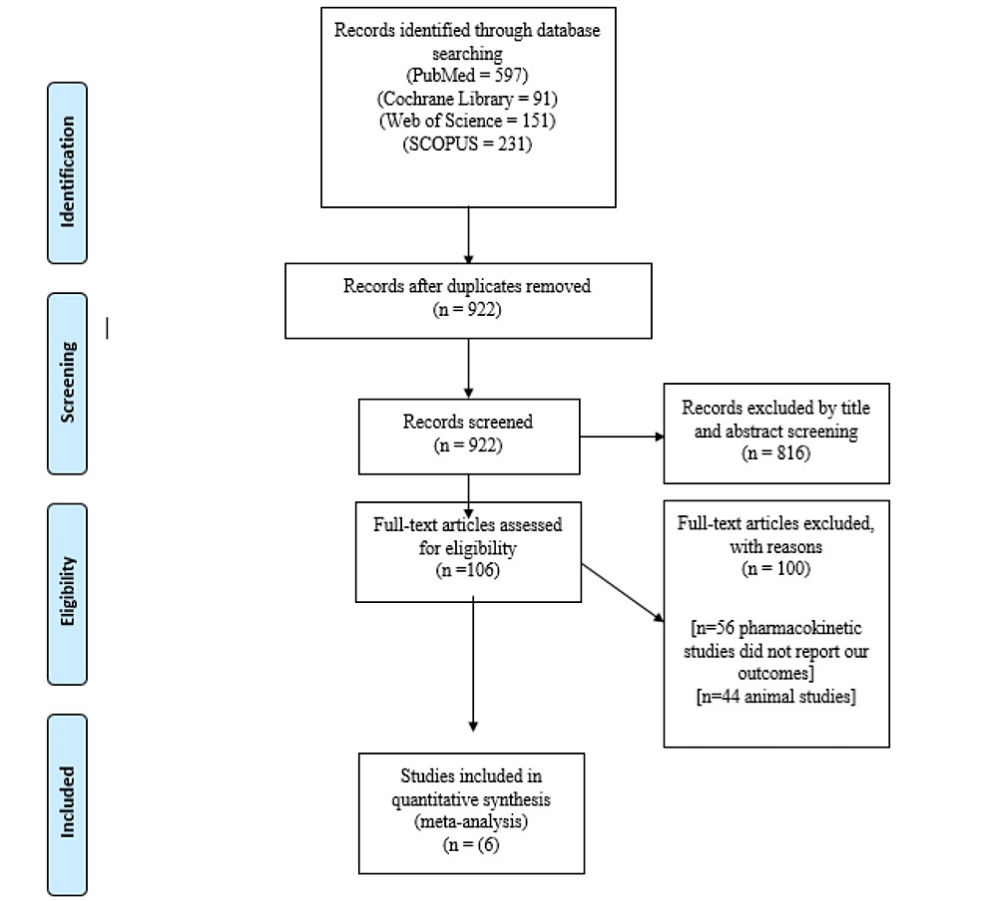
PRISMA flow chart

Our electronic search results are presented in the PRISMA flow diagram (Figure 1). Six papers meeting our eligibility criteria were included [11,12,18-21], encompassing a total of 691 patients with ACL tears. Among them, 343 patients were assigned to the rehabilitation cohort, while 348 patients underwent surgical repair. Both patient cohorts had an average age of 27.4 years. A detailed list of the included trials, along with information on patient age, gender, BMI, Tegner score before injury, nation, and sample size, is provided. Tables 1, 2 show additional baseline data.

**Table 1:** Demographic data about the included participants N, number; SD, standard deviation; BMI, body mass index; NR, not reported

Study ID		Country	Follow-up (years), (median)	Sample size (n)		Age (years), (mean, SD) (median, range)		BMI (mean, SD) (median, range)		Women (%)	
												Surgery	Non-surgery	Surgery	Non-surgery	Surgery	Non-surgery	Surgery	Non-surgery
				1	Reijman et al., 2021 [19]	Netherlands	2	85	82	31.2 (10.3)	31.4 (10.7)	24.3 (3.7)	25 (4.1)	36 (42.4)	31 (37.8)
2	Frobell et al., 2010 [11]	Sweden	2	62	59	26.3 (5.1)	25.8 (4.7)	24.4 (3.2)	23.8 (2.6)	12 (19)	20 (34)
3	Frobell et al., 2013 [12]	Sweden	5	61	59	26.4 (5.1)	25.8 (4.7)	24.5 (3.1)	23.8 (2.6)	12 (20)	20 (34)
4	Sandberg et al., 1987 [21]	Sweden	2	81	76	28 (15-61)	NR	NR	NR	NR	NR
5	Meunier et al., 2007 [18]	Sweden	15	42	52	22 (14-30)	21 (14-30)	NR	NR	11 (25)	21 (37)
6	Tsoukas et al., 2016 [20]	Greece	10.3 (10-11)	17	15	31 (20-36)	33 (25-39)	NR	NR	NR	NR

**Table 2:** Baseline characteristics of knee condition N, number; SD, standard deviation; BMI, body mass index; NR, not reported

Study ID		College education, n (%)		Paid work, n (%)		Tegner score before injury (mean, SD), median (IQR)		ACL injury during sport, n (%)		Objective AP knee instability, n (%)		Meniscal tear	
														Surgery	Non-surgery	Surgery	Non-surgery	Surgery	Non-surgery	Surgery	Non-surgery	Surgery	Non-surgery	Surgery	Non-surgery
		1	Reijman et al., 2021 [19]	30 (35.3)	36 (43.9)	71 (83.5)	64 (78.0)	7.0 (2.3)	7.1 (2.0)	76 (89.4)	71 (86.6)	85 (100)	82 (100)	38 (44.7)	37 (45.1)
2	Frobell et al., 2010 [11]	21 (34)	23 (39)	42 (68)	37 (63)	9 (7-9)	9 (7-9)	62 (100)	57 (97)	61 (98)	58 (98)	39 (63)	30 (51)
3	Frobell et al., 2013 [12]	NR	NR	NR	NR	9 (7-9)	9 (7-9)	NR	NR	60 (98)	58 (98)	NR	NR
4	Sandberg et al., 1987 [21]	NR	NR	NR	NR	NR	NR	NR	NR	NR	NR	NR	NR
5	Meunier et al., 2007 [18]	NR	NR	NR	NR	NR	NR	NR	NR	NR	NR	NR	NR
6	Tsoukas et al., 2016 [20]	NR	NR	NR	NR	NR	NR	NR	NR	NR	NR	NR	NR

Results of Risk of Bias Assessment

**Figure 2:**
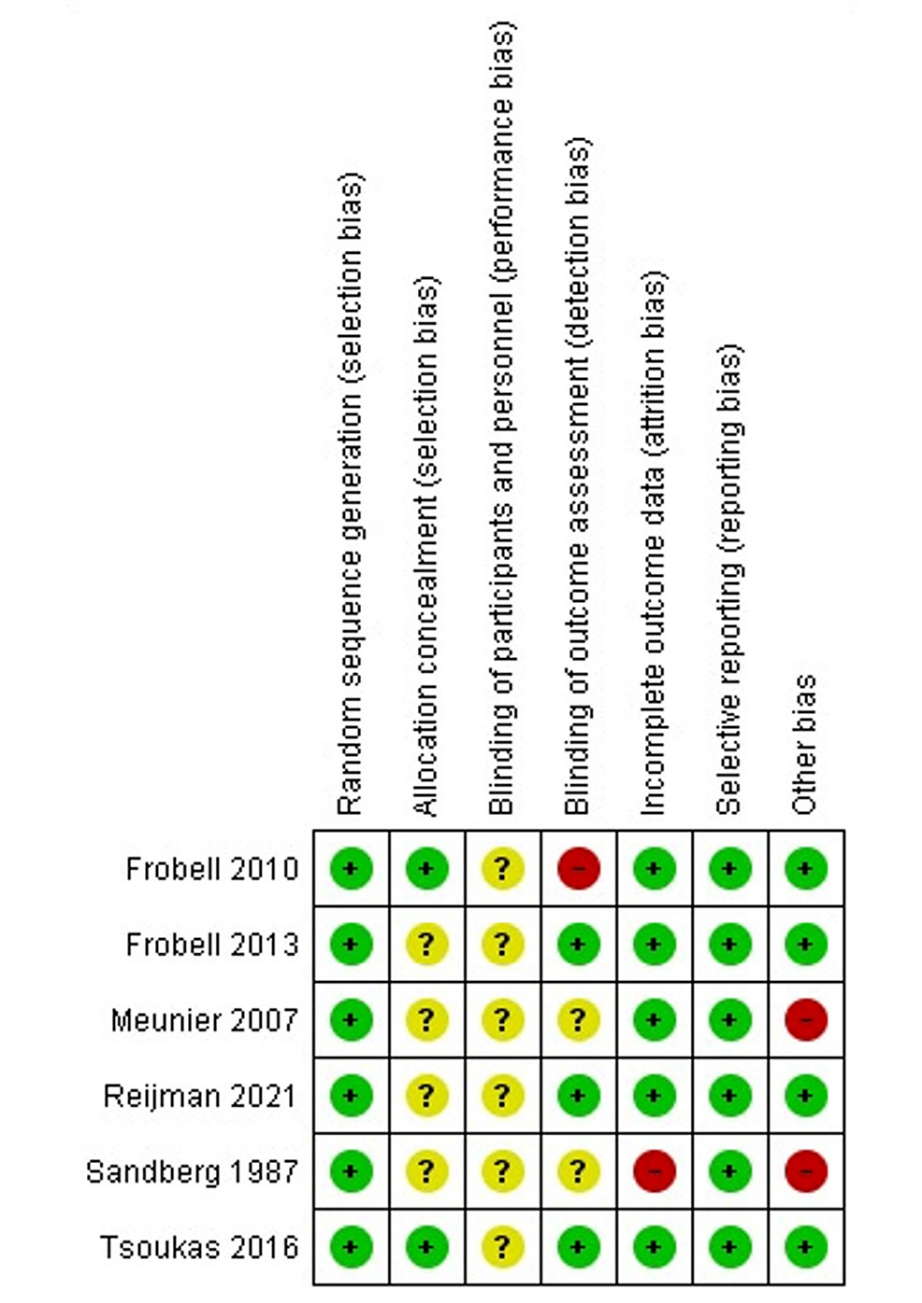
Summary of risk of bias Frobell et al., 2010 [11]; Frobell et al., 2013 [12]; Meunier et al., 2007 [18]; Reijman et al., 2021 [19]; Sandberg et al., 1987 [21]; Tsoukas et al., 2016 [20]

Based on Cochrane’s tool, the included studies’ overall quality was rated as having a moderate risk of bias. We classified all of the trials [11,12,18-21] as low risk because they all reported adequate randomization. Two studies [11,20] were classified as low risk for allocation concealment, whereas the rest of the studies lacked sufficient data and were labeled as ambiguous risks. The remaining quality assessment domains are illustrated in Figure 2.

Analysis of outcomes

IKDC Score

**Figure 3:**
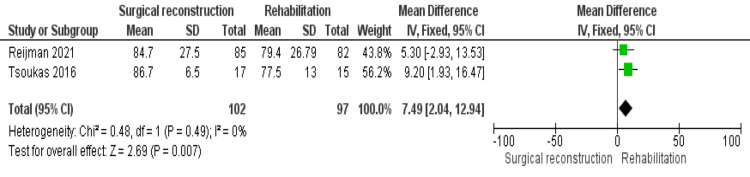
IKDC outcome

Two studies [19,20] reported IKDC scores. The pooled estimate showed no heterogeneity (P = 0.49); I^2^ = 0%. And the overall analysis demonstrated that the IKDC score is significantly higher in the surgical reconstruction cohort than in the rehabilitation cohort (MD = 7.49 [2.04, 12.94], (P = 0.007)) (Figure 3).

KOOS Score

**Figure 4:**
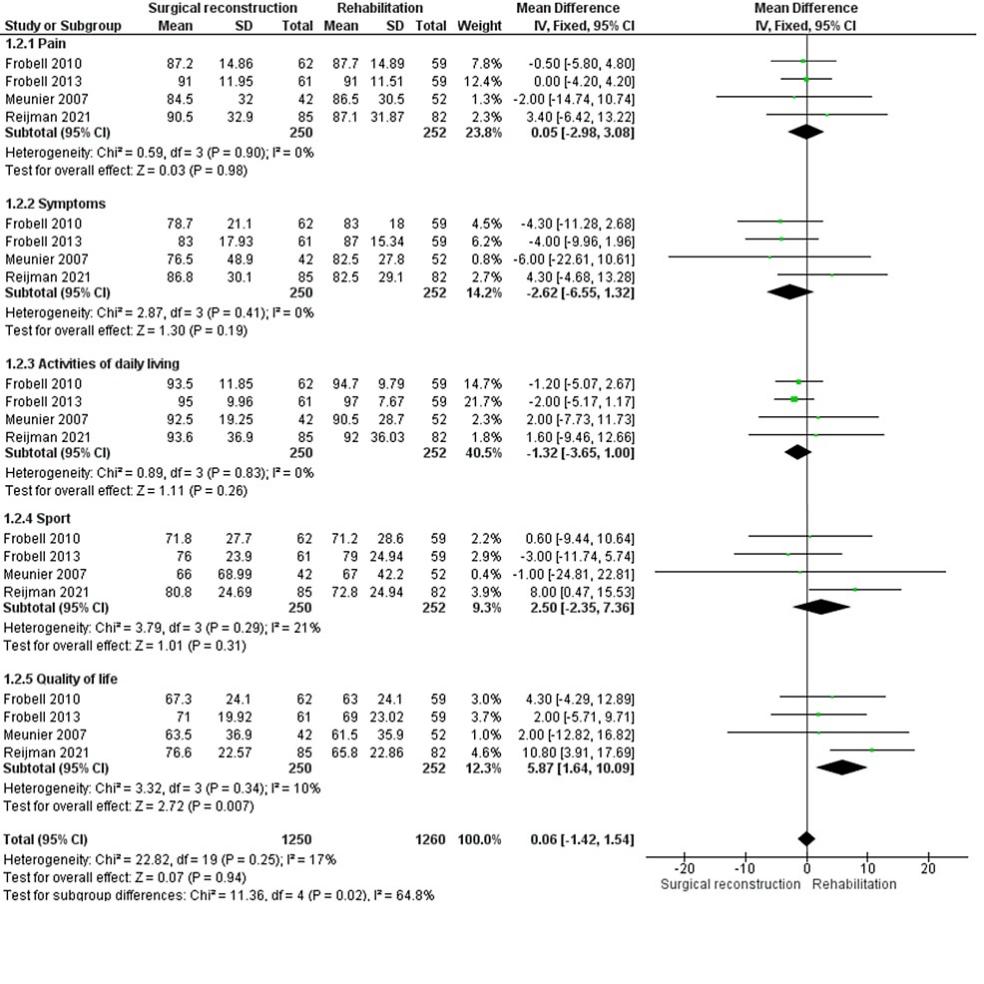
KOOS outcome

KOOS score is reported by four studies [11,12,18,19]. It is a self-reported questionnaire that includes five subscales (pain, symptoms, activities of daily living, sport, and quality of life); therefore, we conducted a sub-analysis of each scale (Figure 4).

Regarding pain, we found no difference between either cohort (MD = 0.05 [−2.98, 3.08], (P = 0.98)). Retrieved data were homogeneous (P = 0.90); I^2^ = 0%.

As for the symptom scale, the overall estimate did not show a significant variation between both cohorts (MD = −2.62 [−6.55, 1.32], (P = 0.19)), and the data were homogenous (P = 0.41); I^2^ = 0%.

Concerning activities of daily living scale, both cohorts showed similar results with no significant variation (MD = −1.32 [−3.65, 1.00], (P = 0.26)). The overall MD was homogenous (P = 0.83); I^2^ = 0%.

Regarding the sports scale, the combined MD showed comparable values in both cohorts (MD = 2.50 [−2.35, 7.36], (P = 0.31)). The analysis was homogenous (P = 0.29); I^2^ = 21%

As for the quality of life scale, we found that patients allocated to the surgical reconstruction cohort reported better results than those who were allocated to the rehabilitation cohort (MD = 5.87 [1.64, 10.09], (P = 0.007)). The overall estimate was homogenous (P = 0.34); I^2^ = 10%.

Lysholm Score

**Figure 5:**
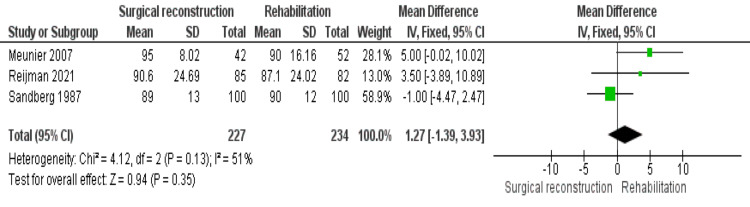
Lysholm score

Three studies [18,19,21] reported the Lysholm score. Data were homogeneous (P = 0.13); I^2^ = 51%. Both cohorts showed similar scores, and the difference between them was not significant (1.27 [−1.39, 3.93], (P = 0.35)) (Figure 5).

Knee Giving Way Occurrence

**Figure 6:**
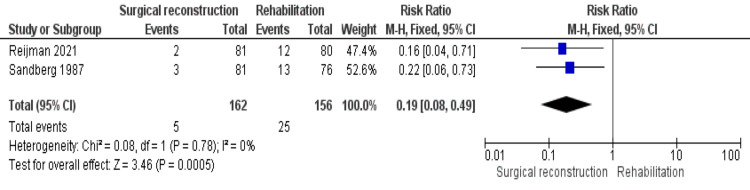
Occurrence of the knee giving way outcome

Only two studies [19,21] reported this outcome. The occurrence of the knee giving way was significantly higher in the rehabilitation cohort (RR = 0.19 [0.08, 0.49], (P = 0.005)). The combined data were homogenous (P = 0.78); I^2^ = 0% (Figure 6).

KT1000

**Figure 7:**
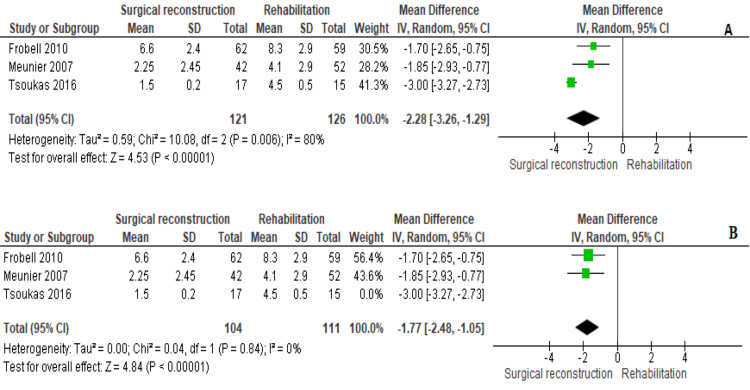
KT1000 outcome

KT1000 was reported by three studies [11,18,20]. The pooled estimate showed that KT1000 was significantly lower in patients allocated to the surgical reconstruction cohort (MD = −2.28 [−3.26, −1.29], (P < 0.001)). We observed heterogeneity among data (P = 0.006); I^2^ = 80%; therefore, we applied a random-effects model. We could solve it by excluding the study by Tsoukas et al., 2016 (P = 0.84); I^2^ = 0%. The MD after heterogeneity was solved, which also showed that KT1000 was lower in the surgical reconstruction cohort (MD = −1.77 [−2.48, −1.05], (P < 0.001)) (Figure 7).

Tegner Score

**Figure 8:**
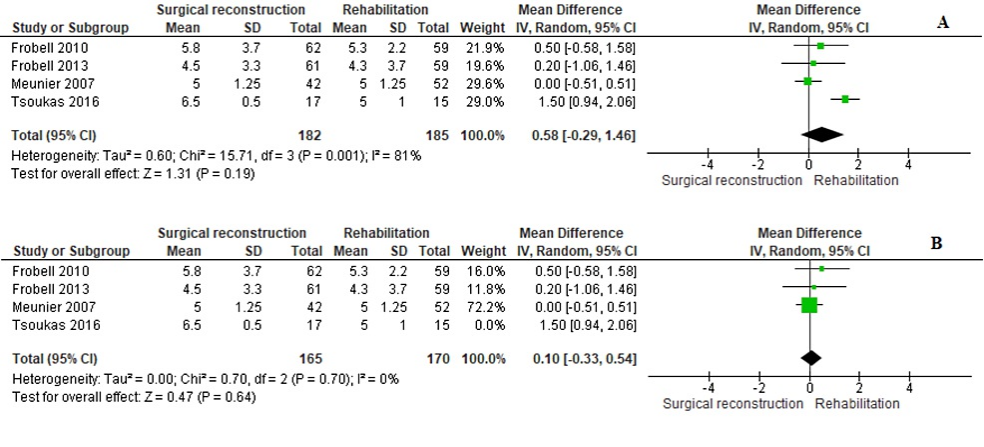
Tegner score

Four studies [11,12,18,20] reported the Tegner score. The combined MD showed similar scores in both cohorts (MD = 0.58 [−0.29, 1.46], (P = 0.19)). However, we observed heterogeneity among studies (P = 0.001); I^2^ = 81%. We could solve this heterogeneity by leaving out the study by Tsoukas et al., 2016 (P = 0.70); I^2^ = 0%. After solving heterogeneity, the overall MD also showed no favoring between both cohorts (MD = 0.10 [−0.33, 0.54], (P = 0.64)) (Figure 8).

Lachman Test

**Figure 9:**
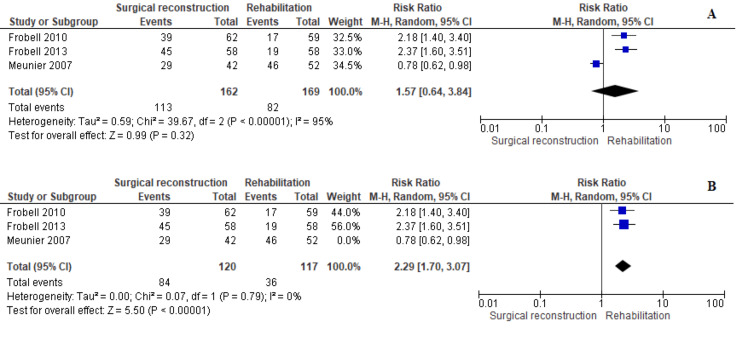
Lachman test

Data on Lachman test outcomes were reported by three studies [11,12,18]. The overall estimate did not favor any cohort (RR = 1.57 [0.64, 3.84], (P = 0.32)). Data were heterogeneous (P < 0.001); I^2^ = 95%; therefore, we tried the random-effects model. Heterogeneity could be solved by excluding the study by Meunier et al., 2007 (P = 0.79); I^2^ = 0%. The combined estimate after solving heterogeneity showed that non-surgical techniques were associated with lower values than the surgical cohort (MD = 2.29 [1.70, 3.07], (P < 0.001)) (Figure 9).

Pivot Shift Test

**Figure 10:**
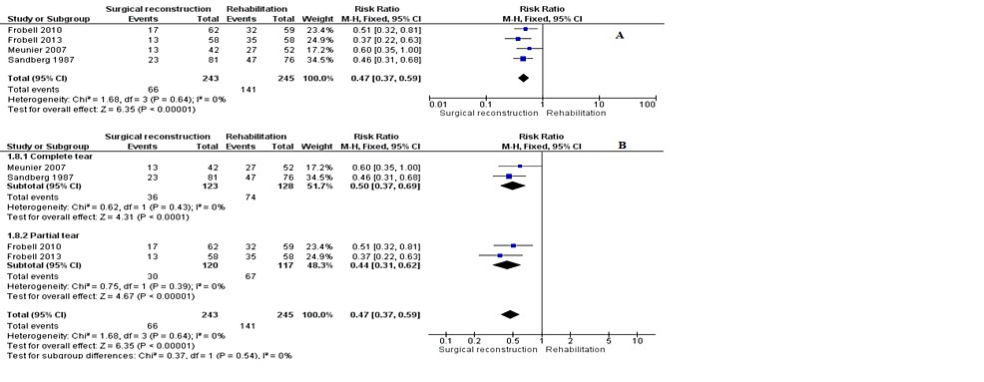
Pivot shift test outcome

Four studies [11,12,18,21] reported data on the pivot shift test. We performed a subgroup analysis according to the severity of ACL injury. The complete tear cohort included two studies (RR = 0.50 [0.37, 0.69], (P = 0.02)). The partial tear cohort also involved two studies (RR = 0.44 [0.31, 0.62], (P = 0.001)). The overall incidence of pivot shift was significantly lower in the surgical cohort (RR = 0.47 [0.37, 0.59], (P = 0.001)). The analysis showed homogeneous data (P = 0.64); I^2^ = 0% (Figure 10).

Reinjury

**Figure 11:**
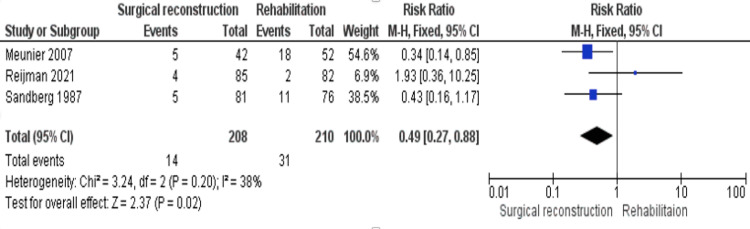
Reinjury outcome

Three trials [18,19,21] reported the reinjury outcome. The incidence of reinjury was significantly lower in the surgical cohort (RR = 0.49 [0.27, 0.88], (P = 0.02)). The analysis was homogeneous (P = 0.20); I^2^ = 38% (Figure 11).

Reoperation

**Figure 12:**
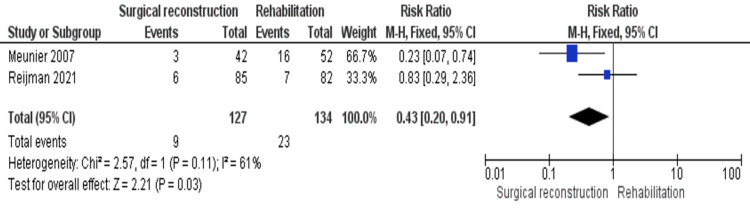
Reoperation outcomes

Two studies reported the need for reoperation [18,19]. The combined RR showed that the need for reoperation was significantly higher in the rehabilitation cohort (RR = 0.43 [0.20, 0.91], (P = 0.03)). The pooled estimate was homogeneous (P = 0.11); I^2^ = 61% (Figure 12).

SF-36 Score

Two trials [11,12] reported an SF-36 score. It consists of two components (physical and mental components); therefore, we conducted a subgroup analysis for each component.

Regarding the physical component, there was no difference between either cohort (MD = 2.31 [−2.17, 6.79], (P = 0.31)). The combined data were homogeneous (P = 0.50); I^2^ = 0%.

**Figure 13:**
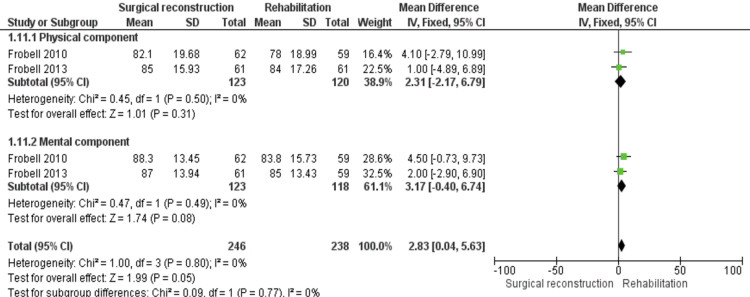
SF-36 score outcomes

Concerning the mental component, pooled estimate showed comparable data in both cohorts (MD = 3.17 [−0.40, 6.74], (P = 0.08)); therefore, we did not face any heterogeneity (P = 0.49); I^2^ = 0% (Figure 13).

Discussion

This meta-analysis encompasses six randomized controlled trials (RCTs) comparing surgical management with rehabilitation in individuals with ACL rupture.

The KOOS is a validated self-reported questionnaire comprising five subscales: pain, symptoms, function in daily living (ADL), sport, and quality of life [22,23]. These subscales assess the short-term and long-term consequences of ACL management through a comprehensive questionnaire comprising 42 items. Patients are assigned a score between 0 and 100, with the score representing the percentage of total achieved points. A score of 0 indicates the poorest knee state, while a score of 100 signifies the absence of any knee problem and the best knee state [24]. Within this meta-analysis, the quality-of-life scale was the sole subscale that demonstrated a significant difference in favor of the surgical cohort. Conversely, the remaining subscales showed similar outcomes between both cohorts. Recent evidence by Reijman et al. [19] comparing surgical and non-surgical management demonstrated that early surgical reconstruction resulted in a higher KOOS score than rehabilitation and optional delayed ACL reconstruction. Frobell et al. conducted two randomized clinical trials in 2010 and 2013, both of which found no significant variation between early reconstruction and rehabilitation regimen, with a primary outcome of KOOS score change over two and five years of follow-up, respectively [11,12]. Irrgang [25] reported comparable mean KOOS scores between both cohorts.

The Lysholm score is a frequently utilized tool to assess the outcomes of ACL reconstruction, encompassing eight subscales and ranging from 0 to 100. Werier et al. [26] reported a mean Lysholm score of 85 (95% CI, 80-90) in the surgical cohort, while the rehabilitation cohort had a mean score of 69 (95% CI, 57-81). The Lysholm score mainly evaluates activities of daily living, with a score below 65 indicating poor outcomes and a score of 95-100 indicating excellent outcomes. Risberg et al. [27] reported poor sensitivity of the Lysholm score in detecting changes over time, but it has shown reliability in patients with various knee disorders [25]. In our meta-analysis, the pooled estimate indicated no significant difference between the surgical and non-surgical cohorts. Reijman et al. [19] found that the early reconstruction cohort achieved a higher Lysholm score than the rehabilitation cohort, while Irrgang [25] reported slightly better results in the surgical cohort compared to the conservative cohort. Conversely, Sandberg et al. [21] reported no difference between both cohorts.

The KT1000 is frequently employed to measure the laxity in the ACL, quantifying the anterior motion of the tibia over the fixed femur [28]. A normal value should not exceed 3 mm, with values exceeding 3 mm indicative of knee laxity [29]. Our analysis favored the surgical cohort over the conservative cohort in this outcome.

The IKDC score is a subjective knee evaluation form comprising multiple items based on patients’ reported outcomes. Our analysis of the IKDC score revealed higher values in the reconstruction cohort.

Patient activity was assessed using the Tegner activity scale, a one-item grading system based on sports activities and work. Scores range from 0 to 10, with 0 indicating a disabling knee injury and 10 signifying high-activity sports. Liow et al. [30] demonstrated the superiority of early reconstruction over late reconstruction, as patients in the early reconstruction cohort returned to a higher activity level than those in the delayed reconstruction cohort, with mean scores of 5 and 4, respectively. However, our analysis did not reveal any significant differences in activity levels between the early reconstruction cohort and the delayed reconstruction and rehabilitation cohorts.

Regarding stability outcomes, the incidences of giving way and the pivot shift test were significantly higher in the reconstruction cohort, while the surgical cohort showed a significantly lower incidence of reinjury and reoperation.

In 2014, Smith et al. [31] published a meta-analysis that found limited differences between isolated ACL reconstruction and rehabilitation concerning clinical outcomes. However, this evidence was based on poor methodology and was insufficient to make a clinical decision regarding the optimal management option. They recommended non-surgical treatment before surgical reconstruction in all patients with ACL rupture. In contrast, Reijman et al. [19] reported that patients in the early reconstruction cohort exhibited significant improvements in knee function, pain perception, and the ability to participate in sports compared to those in the rehabilitation and delayed reconstruction cohort. Furthermore, half of the patients in rehabilitation required surgical reconstruction after unsuccessful rehabilitation. The KANON trial [11,12] reported that the percentage of patients who underwent reconstruction after two years of follow-up was 39%, increasing to 51% after five years of follow-up.

This meta-analysis represents the most recent and highest-quality evidence comparing surgical management with rehabilitation in patients with ACL rupture.

Limitation

The major limitation of our study was the presence of heterogeneous data among studies for some outcomes. However, we addressed this issue using the validated Cochrane's leave-one-out method. The source, if heterogeneity is complex, may be ascribed to variations in sample sizes, patient demographics (e.g., age, gender, body mass index, and duration from injury to intervention), and operative parameters (e.g., type of surgery and surgical expertise of orthopedic surgeon), among others. These variations could have indirectly impacted the extent of heterogeneity and, accordingly, the conclusions of the pooled endpoints.

## Conclusions

In light of this comprehensive meta-analysis, encompassing six meticulously selected RCTs, the evidence firmly supports the comparison of surgical management versus rehabilitation in individuals with ACL rupture. Through rigorous inclusion criteria and meticulous evaluation of diverse outcome measures, this study provides compelling insights into the effectiveness of both treatment approaches, elevating the level of confidence in our findings.

The KOOS and Lysholm score showed no significant differences between cohorts, except for the quality-of-life scale favoring the surgical group. The KT1000 measurement favored surgical management for knee laxity. The IKDC score demonstrated higher values in the reconstruction cohort. Stability outcomes showed higher incidences of giving way and positive pivot shift tests in the surgical cohort but a lower incidence of reinjury and reoperation. These findings align with previous research, emphasizing the efficacy of early surgical intervention for improved knee function, pain relief, and sports participation.

In conclusion, based on the wealth of the most recent and highest-quality evidence available, surgical management, particularly early reconstruction, emerges as the unequivocally superior approach for individuals with ACL rupture. The compelling evidence presented in this meta-analysis, in conjunction with the consistent findings from previous studies, leaves little doubt regarding the efficacy of early surgical intervention in optimizing outcomes for these patients.
